# On Some Points in the Therapy of Chloral Hydrate

**Published:** 1880-02-20

**Authors:** J. W. Hickman

**Affiliations:** Delta, Pa.


### *ON SOME POINTS IN THE THERAPY OF CHLORAL HYDRATE.*

BY J. W. HICKMAN, M.D., DELTA, PA.

My object, in these notes, will be not so much to present new matter in the therapeutics of chloral as to give a bird's-eye view of the present state of our knowledge, as recorded in the literature of our day and as verified in my own experience.

Although known to the chemical world since 1832, chloral was not recognized to possess therapeutic worth until in May, 1869, when Oscar Liebreich, of Prussia, announced that important truth, attested by a well-executed set of experiments.

So far very little of our knowledge of the uses of chloral has been derived from its physiological action; scientific therapeutics has added but a trifling quota to the fund of ascertained facts relative to its practical application in disease. Notwithstanding this, however, we know a great deal of its correct use and much of those conditions contraindicating its administration. For instance, no practitioner who keeps apace with the progress of the day would, for a moment, think of exhibiting chloral where either the respiration or circulation were seriously compromised. It would be absolutely criminal to give it in advanced (or recent, either, for that matter) disease of the lungs where the right heart is being continually overtaxed. Nor would he be less guilty who would think of giving chloral in the sleeplessness of valvular disease of the heart. Yet it has been but a few weeks since I knew a practitioner of a neighboring city to suggest the use of this very drug in precisely such a case as the last-named, with a severe dyspnœa superadded. At this point I must relate a most instructive case recorded by Fothergill: "A patient was taken into the West London Hospital, with emphysema and aortic stenosis. In spite of rest, digitalis, and ammonia, he was liable to attacks of dyspnœa, which had come on *since* his admission into hospital. On searching for an explanation, it was found that the house-surgeon had benevolently prescribed chloral for the sleeplessness complained of. This was at once stopped, and the attacks of dyspnœa never returned, though the man gradually sank." After the principle enunciated above, it seems idle to remark that a fatty heart would not certainly brook even a medium dose of chloral; in such cases paralysis of the heart has occurred before the involvement of the lower centres of the brain. J. Crichton Browne has made a curious observation, and one of no small practical importance. He has ascertained that chloral exerts an effect proportional to cerebral development; that it must be administered most cautiously to the most intellectual. The fact is further borne out in that it takes a larger than ordinary dose of chloral to induce its physiological effects in mocrocephalic idiots. I have seen this latter point abundantly evidenced. This may, perhaps, go to explain some instances of death occurring from a moderate dose—fifteen to twenty-five grains. Again, the matter of bodily temperature should have much to do in determining the proper dose of chloral, for serious symptoms, and even death, have occurred from undue neglect of this consideration. Even so apparently trivial a point as the temperature of the patient's room should be taken into account; for we must remember that the tendency of this agent, besides slowing the action of the heart, is to dilate the peripheral vessels, and thus to increase the heat-losing area of the body. Thus we see that great circumspection should be used in prescribing a remedy of such varying potency as chloral. This care should be taken, not only to avoid death, but that we may derive the most satisfactory and beneficial results from its use.

Standing forth most prominently in the therapeutics of chloral is its value as a sleep-producing agent. The late Prof. Biddle says: "It is a most reliable hypnotic, second only in this particular to opium." Now, from the view of this eminent teacher, the writer feels in duty bound to dissent. It should be recognized as an undisputed fact that there is no soporific agent at all comparable to chloral. Varied in dose, according to rules above laid down, it least frequently disappoints the expectations of the practitioner. It does not induce the swimmy, broken sleep of opium, but quiet, dreamless, and refreshing slumber, strikingly resembling that of health. The patient may be awakened with a confidence that he will at once relapse into forgetfulness. Nor is it, like opium, followed by disagreeable sequences, either cerebral or gastric. On the contrary, so far as the stomach is concerned, digestion is not unfrequently stimulated by its use. It will not, however, exert the slightest soporific influence in painful conditions of the system—it possesses no anodyne properties whatever. When the object is to lull pain and induce sleep, about one-fourth of a grain of morphia sulph. with ten grains of hydrate of chloral will be found a most valuable combination.

Then, too, in conditions of the system where the patient is *too weak to sleep*, anodynes will be found to aggravate rather than secure rest; while in chloral, perhaps, with the bromides given with a little light wine or champagne, we have the most efficient means at our command.

In the sleeplessness due to excessive mental work, chloral exercises a peculiarly happy influence, as the writer has fully verified in his own case while yet a student. When pushed almost to the extreme of nervous and mental exhaustion from this cause, chloral would induce a night of refreshing sleep with an entire renewal of the jaded energies.

Chloral is one of the agents that has seemed to be of service in tetanus. Macnamara lays great stress upon its value, and announces that of twenty cases in India, all traumatic, seventeen recovered by the use of chloral. In large doses it is, perhaps, as effective as any drug we possess. Prof. Gross very accurately expresses the truth, however, when, in summing up the internal treatment of tetanus, he says: "All drugs are, in fact, equally of apocryphal virtue."

In the management of sea-sickness no agent has given such satisfactory results as chloral. The usual dose of about twenty grains is to be given every three or four hours, the patient observing the recumbent posture and taking nourishment in some shape or other.

Bartholow bears personal testimony that there is no remedy now known so efficient in cholera as chloral hypodermically used. The same treatment is also peculiarly effective in cholera morbus. In both conditions the remedy is enhanced in value when combined with a little morphia, as follows: ℞. Chloral hydratis, ʒ iij.; morph. sulph., gr. iv.; aqua laurocerasi, ℥ i. Misce. Sig. From fifteen to thirty minims hypodermically. This injection produces considerable burning pain, and sometimes an indurated lump, but is rarely, if ever, followed by suppuration.

In an extract in The Practitioner for October, 1879, Dr. Curci states that chloral is a most valuable remedy in dysentery. It may be given either by mouth or rectum. When given by the mouth it was found advantageous to anticipate its use by a slight purgative. He is very emphatic in his praise of the remedy, and, judging from its effects upon like lesions on the exterior of the body, together with its ascertained systematic effects, I would strongly advise that the profession give it a trial.

Again, Dr. J. H. Scorff reports four cases where he was successful in removing the vomiting of pregnancy by rectal injections of twenty grains, night and morning. He only found it necessary, in the cases recorded, to administer the remedy three or four times. This also merits remembrance and repetition at our hands.

Chloral is the remedy *par excellence* in the ravings of delirium tremens. Like other drugs, it sometimes deceives us, but in general will be found reliable. Care must be taken in old debauchees that the dose given be not excessive; especially must we be circumspect where there is reason to believe the circulatory system has undergone degeneration of tissue, or rest may be induced which knows no reaction.

Chloral will also be found highly serviceable in certain other conditions of the system attended with delirium and insomnia combined.

It has been recommended and used in puerperal convulsions; but in my opinion is to be absolutely and radically condemned as a *sole remedy*. Too often already has it lulled the physician and friends of the patient into a deceitful security to be trusted further. If there is a truth in the whole domain of medicine, the lancet boldly used is the one and only reliable agent in this condition.

On the contrary, infantile convulsions may frequently be suspended by the discreet use of chloral.

Attacks of laryngismus stridulus are speedily broken up by timely doses of chloral.

Its use has been urged in whooping-cough, but its value is extremely questionable.

Exceptionally good results are obtained in inflammatory and febrile conditions when attended by delirium and wakefulness, with a high temperature. Recently, in a case of typho-malarial fever treated by the writer, chloral was found to control, in a most happy manner, the busy, exhausting delirium attendant thereon. It was administered by enema, and the sleep induced constituted the turning-point in the case. —*Medical Record.*

